# Exploring Monogenic, Polygenic, and Epigenetic Models of Common Variable Immunodeficiency

**DOI:** 10.1155/humu/1725906

**Published:** 2025-04-15

**Authors:** Tayebeh Ranjbarnejad, Hassan Abolhassani, Roya Sherkat, Mansoor Salehi, Fatemeh Ranjbarnejad, Nasimeh Vatandoost, Mohammadreza Sharifi

**Affiliations:** ^1^Department of Genetics and Molecular Biology, School of Medicine, Isfahan University of Medical Sciences, Isfahan, Iran; ^2^Department of Biosciences and Nutrition, Karolinska Institutet, Stockholm, Sweden; ^3^Immunodeficiency Diseases Research Center, Isfahan University of Medical Sciences, Isfahan, Iran; ^4^Cellular, Molecular and Genetics Research Center, Isfahan University of Medical Science, Isfahan, Iran; ^5^Medical Biology Research Center, School of Medicine, Kermanshah University of Medical Sciences, Kermanshah, Iran; ^6^Pediatric Inherited Diseases Research Center, Research Institute for Primordial Prevention of Non-Communicable Disease, Isfahan University of Medical Sciences, Isfahan, Iran

**Keywords:** common variable immunodeficiency, etiology, inborn errors of immunity, primary immunodeficiency

## Abstract

Common variable immunodeficiency (CVID) is the most frequent symptomatic inborn error of immunity (IEI). CVID is genetically heterogeneous and occurs in sporadic or familial forms with different inheritance patterns. Monogenic mutations have been found in a low percentage of patients, and multifactorial or polygenic inheritance may be involved in unsolved patients. In the complex disease model, the epistatic effect of multiple variants in several genes and environmental factors such as infections may contribute. Epigenetic modifications, such as DNA methylation changes, are also proposed to be involved in CVID pathogenesis. In general, the pathogenic mechanism and molecular basis of CVID disease are still unknown, and identifying patterns of association across the genome in polygenic models and epigenetic modification profiles in CVID requires more studies. Here, we describe the current knowledge of the molecular genetic basis of CVID from monogenic, polygenic, and epigenetic aspects.

## 1. Introduction

Common variable immunodeficiency (CVID) is the most prevalent symptomatic entity of inborn errors of immunity (IEI) [1]. CVID patients are characterized by significantly reduced immunoglobulin G (IgG) with IgA and/or IgM in serum and defective antibody production to vaccines because of defective plasma cell differentiation and memory cells. Defects in other additional immune cells have been observed in about 30% of patients with CVID [2, 3]. The clinical phenotype of CVID is heterogeneous in patients. Recurrent respiratory tract infection is a significant manifestation of CVID [4]. Patients with CVID may also exhibit autoimmune disorders, gastrointestinal enteropathies, polyclonal lymphoproliferation, and malignancies [5]. The clinical features could present at any stage in a patient's life, although onset generally peaks at two ages, during childhood (5–10 years of age) and in the third decade (20–30 years of age) [6]. CVID generally has broad and heterogeneous clinical manifestations and immunological characteristics that delay and complicate diagnosis [7]. Diagnosis rates are much higher in families with a high level of consanguinity, multiple affected individuals (with CVID or other types of antibody deficiencies), or founder populations. Some studies on CVID patients have also shown that the consanguinity of parents is associated with early onset of the disease, more severe clinical symptoms, and higher rates of molecular diagnosis [8].

**Figure 1:**
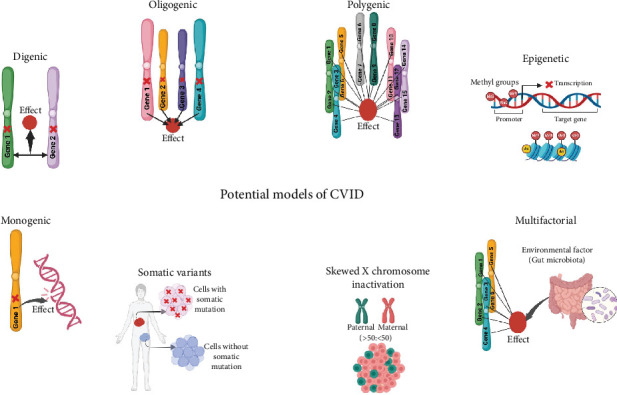
Proposed potential models as the origin of CVID disease. Monogenic: A condition that arises from a pathogenic mutation in a single gene, which can be either dominant or recessive. A monogenic mutation with classical Mendelian inheritance has been detected in a low percentage of CVID patients. Digenic: A genetic condition where mutations in two different genes are necessary to produce a specific phenotype. Oligogenic: A genetic condition that is influenced by minor defects in a limited number of genes. Polygenic: A hypothesized model for unsolved CVID patients is a polygenic model in which a genetic condition is influenced by multiple genes, each contributing a small and cumulative effect. Epigenetic: Another theory for unsolved cases of CVID is epigenetic dysfunction, such as modification in the DNA methylation pattern. Multifactorial: CVID may also be caused by multifactorial inheritance, and the effect of nonhereditary factors (such as gut microbiota) in developing CVID has been identified. Skewed X-inactivation (XCI): Skewed XCI involves the preferential inactivation of one of the two X chromosomes in females. In some women with CVID, the disease results from skewed XCI and highly penetrant gene variants on the X chromosome. Somatic variants: Somatic variants are mutations that occur in somatic cells and can play a significant role in the development of various diseases. A significantly greater prevalence of somatic mutations in the immune cells of CVID patients has been observed than in healthy individuals, potentially contributing to the development of this disorder. Figures were generated with BioRender (https://biorender.com/).

CVID is genetically heterogeneous and observed in sporadic or familial cases with autosomal recessive and autosomal dominant or X-linked inheritance patterns [9]. Identifying gene mutations and diagnosing the molecular basis of the disease are essential steps in elucidating the pathogenic mechanisms underlying CVID and making it possible to provide effective genetic counseling. Furthermore, accomplishing these steps may also introduce molecular targets for further research in personalized medicine for specific patients [10]. Various monogenic mutations in patients with CVID have been reported in recent years, and the list continues to grow. Nonetheless, the cause of the disease is not identified in most cases. The monogenic mutation with classic Mendelian inheritance may be identified in 10%–20% of cases in Western countries or up to 68% of patients in communities with a high level of consanguinity [11]. Although studies have reported several monogenic defects in these patients, more complex genetic models are likely for unresolved nonmonogenic cases [11]. A recent hypothesis proposes that most CVID cases might be due to a complex disease with polygenic inheritance [12]. Consistently, CVID may develop in a multifactorial process by the epistatic interaction of multiple genes and environmental agents, and the role of nonhereditary factors in the development of CVID disease has also been recognized [13]. An alternative hypothesis for cases of CVID with unidentified etiology is that dysfunction in epigenetic regulation may be a contributing factor [14]. Some studies have reported modification in DNA methylation patterns in CVID patients [15, 16]. However, the pathogenic mechanism and genetic basis of CVID are yet to be determined, and the complete identification of the genetic causative factors requires further studies. Proposed potential models as the origin of CVID are depicted in Figure 1.

Identifying the genetic basis of CVID is essential and provides critical knowledge about the genes and pathways related to the development of CVID. Detecting monogenic mutations in CVID enables the development of more comprehensive sequencing gene panels for the genetic diagnosis of CVID. Also, genetic diagnosis of CVID allows the development of targeted therapies and also facilitates carriers and prenatal testing. Recent studies have reported novel mutant causal genes in CVID, but gathering all mutant causal genes for CVID and their associated clinical and immunological characteristics reported in different studies is necessary, and it enables researchers to expedite and enhance their investigation into the genetic basis of CVID.

Evaluating the polygenic aspects of complex diseases by identifying genes and associated variants enables the calculation of genetic risk scores. These scores are derived from various disease-associated variants, which can help predict an individual's susceptibility to complex diseases. Research on rare and common genetic associations enhances our understanding of the genetic landscape of complex diseases, which is vital for understanding the heritability of diseases and the interaction between genetic and environmental influences [17]. Also, understanding epigenetic abnormalities in diseases is of great significance. It enhances our comprehension of disease mechanisms and allows the development of interventions aimed at targeting epigenetic modifications [18]. Recent studies have suggested several potential models to explain the occurrence of CVID, including monogenic, digenic, oligogenic, polygenic, epigenetic, multifactorial, somatic mutations, and skewed X-inactivation (XCI), and there is a need for these studies to be discussed and organized. Although the clinical features and treatment approaches for CVID have been thoroughly documented, significant gaps still exist in our understanding of the mechanisms that underlie the condition. The variability in patient symptoms and responses to treatment indicates that our current knowledge may not completely reflect the complexities of the disorder. This review study aims to explore the monogenic mutations found in CVID disease and their associated clinical and immunological features and the other potential models in the development of this disease.

## 2. Monogenic Inheritance of CVID

Next-generation sequencing (NGS) has instigated a massive revolution in the molecular diagnosis of inherited diseases and the identification of new pathogenic mutations [8]. The use of NGS has led to the detection of a variety of monogenic mutations in patients with the clinical phenotype of CVID, with more than 40 intrinsic B-cell genes linked to this phenotype. These genes are mainly related to the germinal center development and the interaction of B cells with follicular helper T cells and have a crucial function in the signaling pathway of B cells and the process of immunoglobulin (Ig) isotype change; ultimately, their dysfunction results in defective antibody production [19]. Various studies have demonstrated defects in intrinsic B-cell signaling-related molecules at membranous, cytoplasmic, and nuclear molecule levels involved in the pathogenesis mechanism of CVID. Genes involved in monogenic CVID cases are listed based on the latest update from the International Union of Immunological Societies (IUIS) on the classification of CVID phenotype [20] (Tables 1, 2, and 3). Some monogenic mutations have been observed in patients with various clinical presentations, which are related to variable expression or incomplete penetrance and indicate the influence of modifying factors [55]. Also, nongenetic agents like microbial dysbiosis may be involved in the pathogenesis, leading to more significant heterogeneity in CVID disease manifestations [55]. Among the membranous molecules, mutations in the TNFRSF13B gene (encoding TACI) and its ligands, including APRIL and BAFF, are commonly reported in CVID [21]. These molecules participate in the signaling pathway of B cells and Ig class switching [56]. Although studies have shown that mutations of the TNFRSF13B gene (encoding TACI) are reported in about 10% of CVID patients, these mutations have also been observed in healthy people without hypogammaglobulinemia, challenging their pathologic impact and suggesting the involvement of genetic modifiers and environmental agents [7]. Furthermore, defects in membranous molecules of the tumor necrosis factor superfamily, such as the BAFF-R (BAFF receptor encoded by TNFRSF13C) and TWEAK (encoded by TNFRSF12), have been identified in CVID [23, 25]. CD19 is another membranous molecule that acts as a complex with CD81, CD21, and CD225 and promotes the B-cell receptor (BCR) signaling. Defects in this complex lead to defective antigen-dependent signaling of BCRs [30]. Reported in CVID patients are mutations in the components of the CD19 complex (CD19, CD81, and CD21) [28, 29, 31, 57] as well as in membranous costimulatory molecules such as CD20 in CVID [26, 58–61]. These membranous molecules contribute to B cells' survival and developmental process and are possibly involved in the appropriate class-switch recombination (CSR) and isotype selection [62].

Rac2 encodes a component of the Rho family of guanosine triphosphatase (GTPase), which regulates cellular signaling pathways, including cellular migration and actin polymerization [63]. Defects in these cytosolic molecules in CVID patients have been detected by various studies (Table 2). Monogenic mutations have been recognized in other genes that act as cytosolic molecules in the cell, such as POU2AF1 encoding transcriptional coactivator Bob1, which is a specific coactivator stimulating transcription in genes of Ig, and the ARHGEF1 gene, which encodes a guanine nucleotide exchange factor (GEF) and modulates the activities of RhoA GTPases and the RAS superfamily [39, 41, 64, 65]. SH3KBP1 is also a cytosolic adaptor protein identified as a candidate gene in CVID and has a crucial role in vesicle trafficking, remodeling of the cytoskeleton, and downregulation of BCR signaling [40, 66].

Studies have demonstrated that the function, differentiation, and survival of B cells are highly dependent on the phosphoinositide 3-kinase (PI3K) pathways downstream of BCR signaling [67]. Toll-like receptor (TLR) activation, BAFFR-mediated signaling, and chemokine signaling induce PI3K activation, a consequence of which is the promotion of calcium mobilization and nuclear translocation of factor-*κ*B (NF-*κ*B). It has been suggested that the PI3K signaling network also controls the metabolic alterations that activated B cells bring, coping mechanisms for metabolic stress in the plasma cell development processes [67]. The CVID-like phenotype is associated with overactivation of PI3K signaling, usually due to the gain-of-function mutation in the PI3KCD gene or loss-of-function of its inhibitory molecule, that is, PI3KR1 [68]. Leniolisib is an orally administered small-molecule inhibitor that specifically targets PI3K*δ*. It is designed mainly as a targeted therapy for treating activated PI3K delta syndrome (APDS), a rare primary immunodeficiency resulting from mutations that cause hyperactivation of the PI3K*δ* pathway [69]. Recent research, including a Phase 3 randomized controlled trial, has shown that leniolisib effectively reduces lymphadenopathy and restores normal immune cell subsets in individuals with APDS [69]. NF-*κ*B transcription factors (TFs) are nuclear molecules consisting of five subunits that regulate various biological pathways [68]. The NF-*κ*B family plays vital roles in the survival, differentiation, maturation, Ig class switching, and self-tolerance of B cells [68]. NFKB1 gene defect is one of the high-frequency mutations in CVID with an autosomal dominant inheritance [3, 70]. Another subunit of the NF-*κ*B family involved in CVID pathogenesis is NF-*κ*B2, whose mutations interrupt the NF-*κ*B1 pathway by a dominant negative mechanism [44–46, 68, 71, 72]. Studies have shown that unlike NFKB2, NFKB1 pathogenic mutations have been identified in relatives with a milder phenotype and milder antibody deficiency and even in the general population, which may be explained by incomplete penetrance, modifier alleles, and environmental factors. Additionally, patients with NFKB1 mutations have a different age of disease onset and variable expressivity than NFKB2 mutations [43].

**Figure 2:**
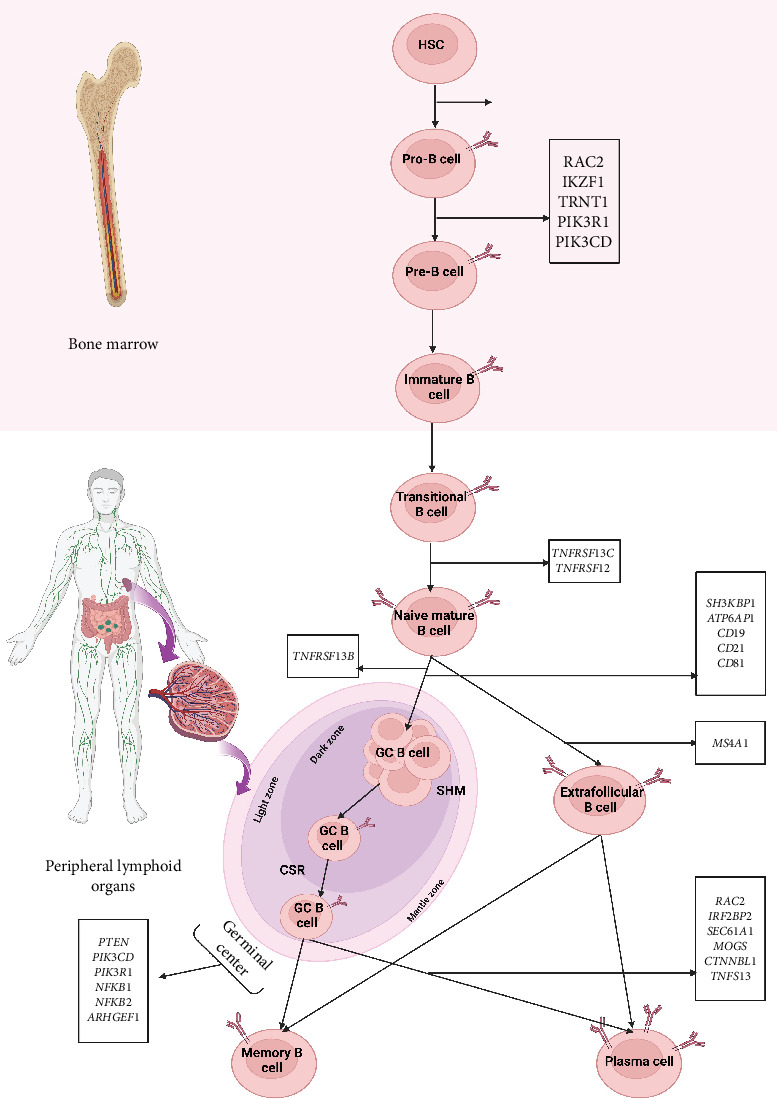
Development pathway of B cell and the role of CVID-related genes in each stage of development. RAC2 is a crucial Rho-GTPase for the process of differentiation of hematopoietic stem cells (HSC) to progenitor B cells (pro-B cells) and then pro-B cells to B-cell precursors (pre-B cells) [79]. Other essential genes during the process of differentiating pro-B cells into pre-B cells are TRNT1, IKZF1, PIK3CD, and PIK3R1 [3]. TNFRSF13C and TNFRSF12 participate in transitional to naive mature B-cell differentiation [80]. Afterward, CD19 complex (CD19, CD81, and CD21), ATP6AP1, and SH3KBP regulate selection and differentiation of naive B cells in peripheral lymphatic tissues [81]. Differentiation of naive mature B cells to germinal center (GCs) B cells and extrafollicular B cells is controlled by TNFRSF13B and MS4A1 gene, respectively [3]. Then, clonal expansion of B cells, CSR, and SHM occurs in the pathway of differentiation of B cells in the GCs, which proteins encoded by PTEN, PIK3CD, PIK3R1, NFKB1, NFKB2, and ARHGEF1 genes make a critical contribution in this pathway [3]. Eventually, the plasma cell differentiation from B cells is controlled by RAC2, APRIL, MOGS, SEC61A1, CTNNBL1, and IRF2BP2 genes [3]. Figures were generated with BioRender (https://biorender.com/).

Other monogenic mutations in genes associated with CVID are nuclear molecules that take part in the signaling pathway of the immune system, mainly B cells, such as PTEN, which is involved in the downregulation of the PI3K/AKT pathway and implicated in regulating cell growth and survival and metabolism [47, 73, 74]; CTNNBL1, which is required for the regulation of multiple splicing events and has a function in somatic hypermutation (SHM) and CSR [48, 75]; IRF2BP2, which is known to repress the functions of the TFs in T cells and regulate the differentiation of plasma blast and memory B cells [3, 51]; TRNT1 (transfer RNA nucleotidyl transferase 1), an essential enzyme for nuclear and mitochondrial tRNA maturation [49, 50, 76–78]; IKZF1, a zinc-finger TF that controls B- and T-lymphopoiesis and function [54]; SEC61A1, a subunit of the Sec61 complex that forms a channel for the translocation of protein in the membrane of the endoplasmic reticulum (ER) and make a critical contribution to related pathways such as antigen processing-cross presentation and plasma cell differentiation [52]; and ATP6AP1, which encodes the component of the vacuolar ATPase complex protein pump and participates in the plasma cell differentiation from B cells and the processing of antigen [3, 53] (Table 3). Tables 1, 2, and 3 present the mutated genes in monogenic forms of CVID based on IUIS classification at the membranous, cytoplasmic, and nuclear molecule levels, along with a description of their genetic, clinical, and immunological features.  Figure 2 depicts the development pathway of B cells and the role of CVID-related genes at each step of the development process.

## 3. Polygenic Inheritance of CVID

**Figure 3:**
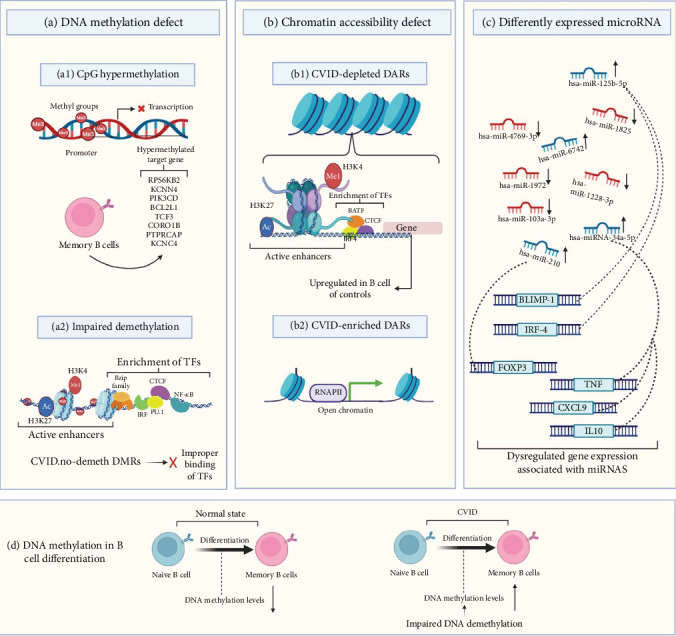
Epigenetic defects in CVID. (a) DNA methylation defect: DNA methylation defect manifest as CpG hypermethylation and impaired demethylation majorly in memory B-cell subsets in CVID. It has been shown that a large number of the nearest genes to these CVID.no-demeth DMRs are upregulated in germinal center (GC) B cells or plasma cells (PCs) from healthy individuals and CVID.no-demeth DMRs are more highly enriched in active enhancer histone marks (H3K4me1 and H3K27ac) in GC B cells. This suggests that the CVID.no-demeth DMRs may contain functional genes relevant to B-cell activity [15, 83]. (b) Chromatin accessibility defect: In CVID, the dysregulation of chromatin accessibility occurs within a specific subset of memory B cells, resulting in some CVID-depleted and CVID-enriched regions. Motif enrichment of several TFs involved in enhancer regulation, such as BATF, IRF4, and CTCF has been described in CVID-depleted topics. Also, the association of CVID-depleted topics with active enhancers has been observed in GC B cells and PCs. These findings suggest that chromatin accessibility defects in CVID affect the regulatory elements essential for B-cell function [83]. (c) Differently expressed miRNA: Recent studies on the expression profiles of miRNAs have revealed that their expression is dysregulated in CVID. Additionally, the upregulation and downregulation of certain genes involved in the immune response have been observed to be associated with the dysregulation of miRNAs [84–87]. The upward arrow indicates an increase in expression, while the downward arrow signifies a decrease. The dotted arrows illustrate the association of some dysregulated genes with miRNAs examined in the study. (d) DNA methylation in differentiation: During the normal differentiation of memory B cells, progressive demethylation takes place. But, in individuals with CVID, memory B cells show impaired DNA demethylation across the entire genome, leading to an increase in DNA methylation levels in these cells [83]. DMRs: differentially methylated regions, DARs: differentially accessible regions, CVID.no-demeth: no demethylated in CVID, CVID-depleted: less accessible DARs in CVID, CVID-enriched: more accessible DARs in CVID. Figures were generated with BioRender (https://biorender.com/).

CVID has a heterogeneous genetic etiology, and as previously mentioned, monogenic mutations are found in a low percentage of patients, although most genetic research into CVID has focused primarily on monogenic subtypes of the disease and more complex genetic scenarios, such as oligogenic or polygenic background, have not received much attention [82].  Figure 3 presents a visual comparison of monogenic, digenic, oligogenic, and polygenic models to enhance comprehension of various inheritance models.

In some CVID patients, digenic inheritance is also proposed, in which pathogenic mutations in two genes are required for the disease occurrence [88]. These cases have been reported, especially in pathogenic mutations of the TNFRSF13B gene [88]. Some pathogenic mutations in the TNFRSF13B/TACI gene have been observed both in healthy people and in patients, which have been suggested to cause the disease, along with pathogenic mutations in other genes in the form of digenic or oligogenic inheritance [88]. In the study by Ameratunga et al., a heterozygous pathogenic mutation in TNFRSF13B and TCF3 gene was introduced as causative mutations that interact in an epistatic way, causing CVID disease in a digenic inheritance model [88, 89].

Several recent studies have investigated CVID from a polygenic aspect using NGS and genome-wide association study (GWAS) platforms and demonstrated that CVID is a polygenic disease in most patients, probably caused by epistatic interactions of multiple genes with cumulative effects [90]. Consistent with this point, CVID can appear at different ages, indicating a gradual and cumulative decline of B-cell function in a hypothesized multifactorial process [15]. Recent studies have investigated the cause of the disease from a polygenic aspect in CVID patients without monogenic mutation. In 2019, Silva et al. described concordant monozygotic twins for CVID who had similar clinical symptoms and immunological profile, and no monogenic mutation was found in them using WES. In this study, the hypothesis of the polygenicity of the disease was investigated using WES data, and analysis of the results revealed a combination of heterozygous and rare deleterious variants, which can indicate the polygenic nature of the disease in these twins. Heterozygous variants were identified in genes of immune-related pathways, such as JUN, JAK3, ICAM1, TLR1, and PTPRC [12].

The purpose of GWASs is to explore the association between single-nucleotide polymorphisms (SNPs) and clinical phenotypes, which comprise the primary method for the genetic investigation of complex diseases [91]. Few GWASs have been performed on patients with CVID as a complex disease, but conducted studies have reported an association between susceptibility loci and CVID disease. Orange et al. conducted the first GWAS and evaluation of copy number variation (CNV) in CVID by genotyping SNPs over the whole genome using the Infinium HumanHap610 BeadChip technology in 363 cases [92]. Their study reported a strong association of ADAM (disintegrin and metalloproteinase) with CVID and confirmed the previously reported association of CVID with the major histocompatibility complex (MHC) region [11, 92]. However, a single causal locus of susceptibility to CVID could not be detected in this study; it was determined that 1000 of the most significant SNPs strongly predict the CVID phenotype and genetic profiling can provide the possibility of predicting cases of CVID [92]. In addition to SNPs being associated with CVID, CNV analysis defined several deletions and duplications also associated with CVID, such as duplication in the ORC4L gene and numerous rare intragenic deletions and duplications, indicating possible multiple genetic mechanisms of CVID. Although none of these structural variants has been functionally confirmed, many SNPs and CNVs have been found exclusively in patients and can be proposed as novel genetic causes of CVID [92]. To prevent population stratification, all the samples included in the analysis, based on the principal components analysis, had white ethnicity, and other samples were excluded. However, the appropriate sample size is necessary to minimize the effect of stratification, which is a challenge to provide in rare diseases. In the most extensive genetic study of CVID to date, 123,127 SNPs were genotyped by Immunochip (Illumina iSelect HD custom genotyping array) in 778 CVID cases and 10,999 controls [93]. This study identified the first significant non-MHC genomic risk source in CLEC16A and confirmed the previously reported MHC associations on chromosome 6p21. Also, in this study, Clec16a knockdown mice showed a decrease in the number of B cells and an increase in IgM, indicating the possible role of the CLEC16A gene in the pathogenic mechanism of immunodeficiency. This study selected patients from five European countries, but ethnicity outliers were removed using PCA (principal component analysis) [93]. In a novel approach, another recent study compared 40 monogenic cases with 43 unsolved CVID patients for multivariant Mendelian randomization (MR). Moreover, deep MHC-Class I and II alleles typing was performed using WES and confirmed by PCR amplicon-based high-resolution typing. The observation of 13 MHC-Class I alleles and 27 MHC-Class II alleles, which are strongly linked to nonmonogenic CVID patients, highlighted the importance of the interactions of environmental antigens with host immunity via the antigen presentation process through MHCs. Moreover, excluding monogenic cases allowed MR analyses to pinpoint significant genetic variations linked to bacterial infections and reduced discrepancies seen in MR analyses of prior GWASs with low pleiotropy, primarily for respiratory tract infections, bacterial infections, and streptococcal infections with distinct patterns compared to MHC haplotype carriers [11]. Although GWAS is an efficient method for detecting genetic variants strongly associated with disease, it identifies only common variants (minor allele frequency (MAF) > 1% or 5%) with small effect sizes, and associations are often not replicated in multiple studies [91]. Because of the limited sample size and statistical power, most of the genetic contribution to the development of complex diseases remains to be discovered even in large meta-analyses of GWAS, and the issue of missing heritability in complex diseases has always been considered in these studies [94]. A possible reason for the problem of missing heritability in GWASs is rare variants that are not evaluated in these studies. Rare variants cause Mendelian disorders, but their role in the occurrence of complex diseases has not been given much attention [94]. Much evidence has shown that rare variants are also associated with complex diseases, but their effect on them has yet to be thoroughly evaluated [95]. The prevalence of CVID varies considerably among populations. According to the distinctive prevalence, polygenicity, and heterogeneity of the CVID disease, the assessment of CVID based on the hypothesis of common disease-common variant (CDCV) is often ineffective. Generally, in IEI patients and CVID in particular, it seems necessary to investigate the cause and pathology of the disease in terms of the hypothesis of a common disease-rare variant (CDRV) [82]. The development of NGS has provided the detection of both common and rare variants. In this method, unlike GWAS, the exome of all genes is examined instead of examining selected candidate genes, which minimizes the chance of missing disease-associated variants [95]. Various studies have used WES data to analyze the association of rare variants with complex immune-related diseases, including Alzheimer's [96], central serous chorioretinopathy [97], amyotrophic lateral sclerosis [98], and inflammatory bowel disease [99]. The association of rare variants with CVID disease was investigated by de Valles-Ibáñez et al. [19]. They examined CVID in monogenic, digenic, oligogenic, and polygenic models by combining exome sequencing with protein–protein interaction (PPI) data. Moreover, for unresolved patients with a pathogenic mutation in a known or candidate gene, CVID was evaluated by digenic and oligogenic models in which heterozygous variants in interacting genes or aggregation of deleterious variants in genes of immune pathways were considered. The authors then proposed a multigene model for CVID and supposed that this disease may be caused by an aggregation of rare variants in genes involved in the same immunological pathway and described the patients with loads of rare variants in genes that have identical functions in the immunology pathway. Eventually, the authors evaluated the association of rare genetic variants with CVID compared with healthy controls using the combined and multivariate collapsing method to identify an excess of CVID cases carrying rare variations. Their aim was not to suggest candidate gene(s) in a specific patient but rather to determine genes enriched for rare genetic variants in cases compared to controls. Data analysis in this study revealed the excess of rare variants in PLCG2, PRKCD, CLEC16A, and DOCK8 genes [19]. To the best of our knowledge, this study is the only rare variant association study in CVID. The control subjects in this study were Spanish, but no information was found on the patient's ethnicity. Also, in this cohort, most patients had early onset CVID (all < 18 years). Studies have shown that CVID patients with early onset disease are more likely to be included in monogenic cases, and other polygenic/complex and epigenetic models are expected in patients with late-onset disease [100]. Therefore, it seems that patients with late-onset disease should also be considered in the population of association study.

Overall, the challenges of diagnosing CVID as a complex or polygenic disorder arise from multiple interconnected factors that complicate the detection of genetic contributions to this condition. Polygenic disorders result from the cumulative impact of numerous genes, with each gene making a minor contribution that complicates the identification of precise genetic variations that cause disease, as the individual impacts of these variations might be minor and sometimes overlooked [101]. Also, the interaction between many genes (epistasis) and between genes and environmental variables adds more complexity to the genetic landscape of complicated disorders. CVID patients present heterogeneous clinical manifestations, which might significantly differ across people and complicate the diagnosis of this condition. Although GWAS have made significant progress in identifying risk alleles, they frequently overlook the complex interactions among numerous risk alleles, and this issue can lead to an overestimation of genetic risk and confuse the interpretation of polygenic risk scores [102]. The reproducibility of genetic risk predictions tends to be low, highlighting the need to integrate genetic data with clinical and lifestyle information for more informative risk assessments [103]. Generally, the practical application of genetic diagnosis in complex disorders like CVID remains restricted and necessitates additional research. Also, the complexity of genetic diagnosis amplifies the challenge of providing genetic counseling to patients [104]. Genetic counselors must elucidate the concept of risk, reduced penetrance, and variable expressivity, which can challenge patients and families to comprehend [104]. Genetic tests for complex diseases frequently produce inconclusive outcomes, which can complicate providing guidance and advice.

## 4. Epigenetic Basis of CVID

Epigenetic modifications reflect the interaction of genes and the environment. Therefore, it is essential to identify epigenetic alterations contribution to the CVID pathogenesis [105]. Some recent studies have emphasized the crucial nature of epigenetic modifications in the pathogenesis of CVID [7]. Epigenetic mechanisms, including DNA methylation, can control the expression of genes without modifying the germline DNA sequence and have a crucial role in immune cell differentiation by regulating gene activity states during cell fate decisions [7]. Despite extensive molecular studies on CVID, few studies have been performed on the epigenome in CVID, and the need for further studies to fully identify epigenome modification in this disease is evident [14]. In this field, recent studies emphasize the critical genes of DNA methylation and their dysfunction that have a great impact on B-cell function, including DNMT3B and ZBTB24. Thus, a defect in these DNA methylation regulators restricts the activation and differentiation of B cells resembling the CVID phenotype [106].

Rodríguez-Cortez et al. [15] presented the first report of alterations in the DNA methylation profile of CVID in a case of discordant monozygotic twins using Infinium HumanMethylation450 BeadChips. They identified CpG hypermethylation with failed demethylation and the consequential reduction in gene expression in critical B-lymphocyte genes in B cells of CVID case compared to B cells from the healthy sibling. This observation included impaired demethylation of the RPS6KB2, KCNN4, PIK3CD, BCL2L1, TCF3, CORO1B, PTPRCAP, and KCNC4 genes. In continuation of this study, additional evaluation of DNA methylation patterns in 16 CVID patients and 12 healthy controls has shown increased DNA methylation levels in the selected genes in switched and unswitched memory cell subsets but normal levels of methylation in the naive B cell of CVID cases, suggesting impaired DNA demethylation and then overexpression of related genes in the transition of naive to memory B cells in CVID [15]. In another study by these authors on CVID discordant monozygotic twins (same twin pairs as in the previous study) and a cohort consisting of 10 patients diagnosed with CVID and 10 healthy individuals serving as controls, multiomics datasets of single-cell (transcription and chromatin accessibility and DNA methylation) were produced. Their data show widespread modifications in chromatin accessibility and DNA methylation in memory B-cell CVID cases that affect the transcriptome of memory cells. These transcriptional and epigenetic changes indicate defects in the communication of immune cells, which may be the cause of defective immune responses in CVID. In parallel, whole-genome sequencing (WGS) was carried out using a NovaSeq sequencer (Illumina, California, United States) to identify the possible genetic basis of CVID discordance in the twins. The results of WGS revealed no differential pathogenic mutations between the twins. The results also revealed that genetic variants shared by both twins in CVID-related genes such as TNFRSF13B, NFKB2, CR2, and CD19, which strengthens the hypothesis that another change, such as environmental exposure and epigenetic modifications, may be critical factors in the discordant phenotype of these twins [83].

Tallmadge et al. [107] described aberrant epigenetic regulation using an equine CVID model. In their study, hypermethylation of the PAX5 gene, an essential regulatory protein in the development of B cell, was observed by bisulfite pyrosequencing. Additionally, a bone marrow transcriptional analysis by RNA sequencing using the Illumina HiSeq 2000 indicated the downregulation in the expression of genes that regulate the differentiation of B cells, especially PAX5. According to all the evidence provided, aberrant epigenetic regulation caused silencing of the PAX5 gene and the occurrence of late-onset and sporadic CVID manifestations in an equine model [107]. In another study, specific CpG sites related to critical genes in the activity of B cells and differentiation that undergo significant demethylation in healthy individuals were selected and evaluated by bisulfite pyrosequencing in memory and naive B-cell subpopulations from CVID cases and healthy individuals. Defective demethylation was observed in the specific CpGs in genes controlling specific B-cell pathways and especially in B-cell signaling in memory B cells. The level of impairment in demethylation was proportional to the decrease in the amount of memory B cells [16]. Research has shown that in memory B cells of patients with CVID, CpG sites located in regulatory elements of genes key to B-cell function are hypermethylated. In contrast, methylation levels of these sites are reduced from the transition of naive to memory B cells in healthy subjects [16].

An abnormal DNA methylation pattern of B cells has been considered to be a key characteristic of the dysregulation of the B cell in CVID. Above all, the severe memory B-cell deficiency in patients with CVID is often associated with impaired demethylation of some genes, indicating the potential role of their defective demethylation in impairing memory B-cell generation. These results underscore the potential of reduced demethylation as a promising area for future research into the defects in the maturation of B cells in CVID patients [16]. DNA methylation and chromatin accessibility abnormalities in switched memory B cells of individuals with CVID will likely influence gene expression [83]. In CVID, switched memory B cells exhibit significant variability in their DNA methylation patterns. This aberrant methylation is associated with the active demethylation of TFs that are crucial for B-cell differentiation and activation, including NF-*κ*B, members of the Bzip family (such as Fra2, Fosl2, JUNB, and BATF), CTCF, IRF, and PU.1, which ultimately disrupts their ability to bind to DNA effectively [83]. Also, the analysis revealed significant enrichment of these TFs that are involved in B-cell differentiation and function within the differentially methylated regions (DMRs) that do not undergo the expected demethylation during B-cell differentiation in CVID patients [83]. Additionally, there has been identified dysregulation of genes such as CD70, CD40, NFKB2, ICAM1, and CCL17 in activated memory B cells, particularly in regions of hypermethylated DNA [83].

MicroRNAs (miRNAs) play a crucial role in various biological processes and are essential for immune function. They regulate the survival and activity of B and T lymphocytes and are involved in the epigenetic regulation of gene expression during B-cell differentiation and maturation. Therefore, the evaluation of miRNAs as important factors in the development of the disease has been of interest in CVID research [108]. The study on unsolved CVID patients indicated increased expression of miR-210 in T lymphocytes [84]. Investigation of miR-125b-5p (effect on BLIMP-1 and IRF-4 gene expressions) in unsolved CVID patients indicated that miR-125b-5p overexpression may impair B cell's terminal differentiation [87]. Other research in this field has also highlighted that the deregulation of miR-155 and miR-142 could lead to CVID phenotypes in animal models, and their dysregulation has been observed in comorbidities of CVID in humans, including cancer and autoimmunity [109]. A recent investigation discovered five miRNAs that exhibit differential expression in patients with CVID after their initial Ig replacement therapy. The identified miRNAs are hsa-miR-6742, hsa-miR-1825, hsa-miR-4769-3p, hsa-miR-1228-3p, and hsa-miR-1972. Importantly, all of these miRNAs were downregulated following the Ig infusion, except for miR-6742, which showed an increase in expression [85]. Recent research indicates that miRNAs are correlated to the inflammatory response in CVID and may influence its clinical manifestations [86]. A 2024 study involving CVID patients assessed circulating miRNA levels and plasma inflammatory proteins, comparing these with healthy controls [86]. The findings revealed that miRNA-34a levels were associated with 11 inflammatory proteins, including TNF, CXCL9, and IL10 suggesting a biological association between them. Additionally, a negative correlation was observed between the levels of miRNA-34a and the count of naive CD4 T cells in individuals with CVID [86].  Figure 3 describes epigenetic defects in CVID including DNA methylation defects, chromatin accessibility defects, and dysregulation of miRNAs.

## 5. Other Potential Models in CVID

### 5.1. Multifactorial

Studies have shown the potential contribution of various environmental factors such as microbiota alterations, infectious agents, and lifestyle factors in the development of CVID (Figure 1). Alterations in the composition and function of the gut microbiome, known as dysbiosis, have been identified as significant factors contributing to intestinal disorders and inflammatory conditions [110]. Studies show that people with CVID frequently display alterations in their gut microbiota, notably a decrease in bacterial diversity [110]. This dysbiosis can worsen immune dysfunction, as a reduced variety of microbial populations may hinder immune regulation and heighten the risk of infections. Recent studies have shown that patients with CVID, in comparison to healthy controls, exhibited a disturbed oropharyngeal microbiota, characterized by decreased diversity in the respiratory microbiome and an increase in potentially pathogenic bacteria [111].

Environmental exposure to different pathogens can affect how CVID manifests clinically. Patients with CVID frequently experience recurrent infections. These infections can result in chronic issues such as bronchiectasis or lung fibrosis, which further complicate the condition [112].

To the best of our knowledge, research has primarily focused on the role of the microbiota as an environmental risk factor in the multifactorial disease model of CVID. Some studies have identified factors like hematopoietic stem cell transplantation (HSCT) and the use of specific medications, such as rituximab, as potential risk factors for hypogammaglobulinemia [113, 114]. It is plausible to hypothesize that these factors may contribute to the development of antibody deficiency and a CVID-like phenotype, and thus, it is recommended that they be explored in future research.

### 5.2. Somatic Variants

Somatic variants are changes in the DNA that occur after fertilization, during embryonic development, or lifetime of individuals [115]. These mutations give rise to a condition called mosaicism, in which different cells within the same person have distinct genetic makeups. This can lead to diverse phenotypic expressions across various tissues [115]. Throughout a person's life, these mutations accumulate due to factors like errors in DNA replication and inherent cellular and environmental influences [116]. While the literature has extensively documented somatic mutations that can mimic or compensate for IEIs across various genes, the investigation of somatic variations in CVID is still in its early stages [90]. Disease-causing somatic mosaicism has been found in genes that are known to cause germline IEI, such as FAS, as well as in genes that do not have significant corresponding germline diseases, like UBA1 and TLR8. Postzygotic variants in genes linked to CVID, including PIK3CD, STAT3, and BTK, have been discovered in affected families, indicating the existence of somatic or gonosomal mosaicism [117]. A study found somatic mutations in CD4+ and CD8+ T cells in 65% of individuals with CVID and other immunodeficiencies, compared to 48% of healthy controls. According to the study's findings, clonal T-cell populations may affect B-cell activities and result in particular clinical issues [118]. In addition to IEI, somatic mosaicism has gained recognition as a significant factor in several autoinflammatory disorders, especially those involving particular genetic variants [119]. Key genes associated with this phenomenon include NOD2, IL6ST, NLRC4, JAK1, and KRAS, all of which are linked to essential pathways for immune regulation and inflammation [120]. Studies have shown that somatic variants in STAT5B and TET2 may be associated with developing autoimmune disorders, lymphoproliferative conditions, and malignant transformations [118, 121]. Also, somatic pathogenic variants in the GATA2 gene have important consequences for patients with immunodeficiencies, especially those who exhibit deficiencies in B and NK cells and have a history of opportunistic infections [121]. Figure 1 illustrates the role of somatic mutations as a possible contributing factor to the development of CVID disease.

### 5.3. Skewed XCI

Skewed XCI refers to a situation in which one of the two X chromosomes in females is preferentially inactivated over the other [122]. This leads to an unequal distribution of active X chromosomes among cells. Such a phenomenon can have important consequences for several health issues, especially those associated with X-linked genes [122]. Skewed XCI can worsen the severity of X-linked disorders. Normally, the recessive mutation is not expressed on the inactive X chromosome, because the inactivation is balanced [123]. However, when skewing occurs, it may increase the expression of deleterious alleles, which can lead to more severe symptoms in carriers of these mutations [124]. Evidence has shown the association of skewed XCI with autoimmune diseases such as systemic lupus erythematosus (SLE) [125]. Studies have suggested that altered patterns of XCI in immune cells may contribute to the sex bias observed in autoimmune conditions, as females are disproportionately impacted relative to males [125]. A 2024 study found that in some women with CVID, the disease is caused by skewed XCI and highly penetrant gene variants located on the X chromosome [123]. These findings suggest that skewed XCI could be an important factor in the complex genetics of CVID. Numerous genes associated with antibody deficiency are found on chromosome X, including BTK, IL2RG, SASH3, CD40LG, IKBKG, SH3KBP1, SH2D1A, FOXP3, ATP6AP1, and MAGT1 [123]. Therefore, testing for XCI status can be suggested on female patients suspected of harboring mutations in these genes. The possible contributory or causal impacts of these variants on CVID can be clarified by identifying a skewed XCI pattern with dominant expression of the mutant allele [123]. As a result, skewed XCI could be considered a significant component of the complex genetic landscape of CVID. Figure 1 illustrates the role of skewed XCI as a possible contributing factor to the development of CVID disease.

## 6. Conclusions

A low percentage of CVID patients have monogenic mutations with Mendelian inheritance. In unresolved CVID patients, polygenic, epigenetic, and multifactorial models have been proposed in most studies as models of disease development. Limited studies have reported that digenic and oligogenic inheritance as well as somatic mutations and skewed XCI contribute to the CVID disease. So, it seems that in nonmonogenic cases, CVID most often be a complex disease in which different gene variants participate with cumulative effects, with or without environmental influences. The challenges of diagnosing CVID as a complex or polygenic disorder arise from multiple interconnected factors that complicate the detection of genetic contributions to this condition. Therefore, it is recommended that the polygenic investigation of unresolved CVID patients, especially the rare variant association test, be prioritized in future studies. Although the findings confirm the polygenic nature of the disease, the variants and genes involved in the polygenic model and how they affect are still unknown and require additional studies. Polygenic studies in CVID have shown the association of variants in some genes related to the immune system, such as the MHC locus, with CVID. These findings show that the interaction of deleterious variants in the immune system–related genes and probably the interaction of environmental factors may lead to the development of CVID in complex cases. However, genetic diagnosis in complex disorders such as CVID is yet limited and requires more research. Precise genetic risk calculation is impossible, complicating the issue of providing genetic counseling to CVID patients. In nonmonogenic cases, prenatal and carrier testing is challenging and not applicable, which complicates the management of this disease. In this study, we summarized the potential effective variants associated with CVID, but future studies are necessary for precise genetic risk assessment. On the other hand, epigenetic modifications which are affected by environmental factors such as infections may have a key role in the pathogenesis of complex diseases with a wide spectrum of clinical presentation and ages of onset. Studies have revealed epigenetic modifications in the B cells of some CVID patients, but the findings are not enough; a more complete understanding of the epigenetic modification pattern in this disease requires an investigation of high proportions of patients. Furthermore, due to the involvement of other immune cells such as T and NK cells in CVID disease, in addition to B cells, the examination of epigenetic modifications and DNA methylation patterns in these cells seems to be necessary. Considering the effect of the environment on epigenetic modifications, it is suggested to consider the potential role of environmental factors in CVID disease by the mechanism of affecting epigenetic modifications in future studies. Also, due to the reversibility of epigenetic modifications, the possibility of using treatments targeting epigenetic modifications should be considered in future research. In conclusion, a comprehensive understanding of monogenic, polygenic, and epigenetic modifications in CVID can provide insights for future research and development of targeted treatments and management strategies.

## Figures and Tables

**Table 1:** Monogenetic defects of cell membrane molecules in CVID.

**Genetic defect**	**Disease**	**Mutation type**	**Inheritance pattern**	**Disease onset**	**Clinical manifestations**	**Immunological features**	**Ref**
TNFRSF13B (TACI)	TACI deficiency	LOF	AR/AD	Early childhood to adulthood	AIHA, ITP, ILD, AIE LAD, GD, GIs, BLH, splenomegaly, granulomata, AI, cancer	↓IgG, nl or ↓IgM, nl or ↓ IgA, ↓ post-vaccination antibody levels, nl or ↓ or ↑ total B cells, nl or ↓ MBCs, nl or ↓ Treg cells, nl or ↓ total/CTLs/Th cells, ↓ or nl CD8+ and CD4+ MTC or TN	[21, 22]

TNFRSF13C (BAFF-R)	BAFF receptor deficiency	LOF/GOF	AR/AD	Neonatal-onset to late adulthood	Diarrhea cholangitis, sacroiliitis, AI, splenomegaly, FTT, BLH, granulomata, GIs	↓ IgG, nl or ↓ IgM, nl or ↓ IgA, ↓ post-vaccination antibody levels, nl or ↓ total B cells, nl or ↓ MBCs, nl or ↑ TrB cells, nl total T cells	[23, 24]

TNFRSF12 (TWEAK)	TWEAK deficiency	LOF	AD	Neonatal-onset	Meningitis, warts, AI neutropenia and thrombocytopenia, osteomyelitis	nl or ↓ IgG, ↓ IgG2, ↓ IgA, ↓ IgM, ↓ post-vaccination antibody levels, ↑ NBCs, ↓ or nl total B cells, ↓ MBCs, nl or ↑ total T cells, ↑ CTLs, nl Th cells	[25]

MS4A1 (CD20)	CD20 deficiency	LOF	AR	Neonatal-onset	RI	↓ IgG, nl IgA, nl IgM, ↓ post-vaccination antibody levels, nl total B cells, ↓ MBCs, nl subsets, and total T cells	[26]

TNFS13	APRIL deficiency	LOF	AR	Childhood	RVI, alopecia areata	↓ IgG, ↓ IgM, and ↓ IgA, nl total B cell, ↑ MZBCs, ↓ switched MBCs	[27]

CD19 complex (CD19, CD81, and CD21)	CD19 deficiency	LOF	AR	Neonatal-onset to early childhood	Conjunctivitis, GIs, bronchiectasis, skin infections, IgA nephropathy, glomerulonephritis	↓ IgG, ↓ or nl IgM, ↓ or nl IgA, ↓ post-vaccination antibody levels, nl total CD20+ B cells, ↓ BCR signaling, ↓ MBCs, ↓ CD21 expression, nl T cells	[28, 29]
	CD81 deficiency	LOF	AR	Neonatal-onset	SIRS, glomerulonephritis with end-stage renal disease, AI thrombocytopenia	↓ IgG, nl IgM, ↓ nl IgA, ↓ post-vaccination antibody levels, nl total CD20+ B cells, ↓ MBCs, nl T-cell subsets	[30]
	CD21 deficiency	LOF	AR	Childhood	Myalgia, rigidity, splenomegaly, chronic diarrhea	↓ IgG, ↓ or nl IgM, ↓ IgA, ↓ post-vaccination antibody levels, nl total CD19+ B cells, mildly, ↓ MBCs, nl T cell	[31]

*Note:* Recurrent infections, especially recurrent respiratory tract infections, were similar clinical features across various genetic categories and are not listed in the table. Ref, references; ↑, increased; ↓, decreased; nl, normal; Teff, effector T cells; Treg, regulatory T (CD4 + CD25 + FoxP3+).

Abbreviations: ACD, allergic contact dermatitis; AD, autosomal dominant; AI, autoimmunity; AIE, autoimmune enteropathy; AIHA, autoimmune hemolytic anemia; AIN, autoimmune neutropenia; AIT, autoimmune thyroiditis; AR, autosomal recessive; BCR, B-cell receptor; BLH, benign lymphoid hyperplasia; CTLs, cytotoxic T lymphocytes; DLE, discoid lupus erythematosus; DN, double negative; EBV, Epstein–Barr virus; FTT, failure to thrive; GD, granulomatous disease; GIs, gastrointestinal infections; GLILD, granulomatous lymphocytic interstitial lung disease; GOF, gain-of-function; HLH, hemophagocytic lymphohistiocytosis; HSV, Herpes simplex virus; IBD, inflammatory bowel disease; Ig, immunoglobulin; ILD, interstitial lung disease (including lymphocytic interstitial pneumonitis, interstitial pneumonitis, autoimmune infiltrative pulmonary disease, and cryptogenic organizing pneumonia); ITP, idiopathic thrombocytopenic purpura; LAD, lymphadenopathy; LPD, lymphoproliferative disorders; LOF, loss-of-function; MBCs, memory B cells; MZBCs, marginal zone B cells; NKCs, natural killer cells; NKT, natural killer T; RI, recurrent infections; RVI, recurrent viral infection; SIRS, systemic inflammatory response syndrome; Th, T helper (CD4+); TN, naive T cells; T1DM, Type 1 diabetes mellitus.

**Table 2:** Monogenetic defects of cytosolic molecules in CVID.

**Genetic defect**	**Disease**	**Mutation type**	**Inheritance pattern**	**Disease onset**	**Clinical manifestations**	**Immunological features**	**Ref**
PIK3R1	APDS2	LOF	AD	Neonatal-onset to childhood	Bronchiectasis, CMV/EBV viremia, GIs, conjunctivitis, FTT, AI, cancer, LPD, hepatosplenomegaly	↓ IgG, ↓ IgA, ↓ or nl or ↑ IgM, ↓ postvaccination antibody levels, ↓ or nl total B cells, ↓ or nl MBCs, nl or ↑ TrB cells, nl or ↑ total T cells, ↓ or nl CD4+ T cells, ↓ or nl or ↑ CD8+ T cells, nl or ↑ Treg cells, nl DN T cells, ↓ or nl NKCs	[32, 33]
PIK3CD	APDS1	GOF	AD	Neonatal-onset to childhood	GIs, AIHA, AIT, ILD, GHD, GN, skin infections, FTT, CMV/EBV viremia, bronchiectasis, AI, LPD, warts, deep abscesses, cancer, hepatosplenomegaly	nl or ↓ or ↑ IgG, ↓ or nl or ↑ IgM, nl or ↓ IgA, ↓ post-vaccination antibody levels, ↓ or nl total B cells, nl or ↓ MBCs, ↓ or nl NBCs, nl or ↑ TrB cells, ↓ or nl CTLs/Th and total T cells, ↓ or nl CD4+/CD8+ TN, nl or ↑ CD8+/CD4+ MTCs, ↓ or nl or ↑ NKCs, ↓ or nl or ↑ NKT cells	[34–36]
MOGS	Mannosyl-oligosaccharide glucosidase deficiency	LOF	AR	Neonatal period to childhood	Dysmorphic features, recurrent fractures, neurologic disorders, sensorineural hearing loss, DD, otitis media	↓ IgG, ↓ IgM, ↓ IgA, ↓ post-vaccination antibody levels, ↓ CD19 + IgG+, nl plasma cells, ↑ immature B cells, ↑ TrB cells, ↓ neutrophils	[37]
Rac2	RAC2 deficiency	LOF	AR	Neonatal-onset to childhood	FTT, BLH, glomerulonephritis, allergy, coagulopathy, bronchiectasis, arthralgia, AI endocrinopathy	↓ IgG, ↓ IgA, ↓ IgM, ↓ post-vaccination antibody levels, ↓ total B cells, ↓ TN cells (CD4+/CD8+), ↓ CD4+ RTEs, ↓ Treg cells	[38]
ARHGEF1	ARHGEF1 deficiency	LOF	AR	7 and 10 years old	Varicella-Zoster virus, bronchiectasis, mucoepidermoid carcinoma	↓ IgG, nl IgA, ↓ IgM, ↓ post-vaccination antibody levels, ↓ total B cells, ↓ MBCs, ↑ TrB cells, remarkably ↓ MZBCs, remarkably ↓ switched MBCs, ↓ isohemagglutinin titer, TN cells (CD8+)	[39]
SH3KBP1	SH3KBP1 deficiency	LOF	XL		RBI	↓ IgG, ↓ IgM, ↓ IgA, ↓ post-vaccination antibody levels, nl B-cell subsets, ↓ switched MBCs, nl T-cell subsets	[40]
POU2AF1	BOB1 deficiency	LOF	AR	18 months	Viral infection of the CNS.	Agammaglobulinemia: very low IgG, IgA, IgM, ↓ switched MBCs, ↑ atypical MBCs, ↑ NKCs	[41]
PIK3CG	PIK3CG deficiency	LOF		Childhood	Cytopenia, T lymphocytic pneumonitis and colitis, splenomegaly, LAD, HLH-like	↓ IgG, ↓ IgA, ↓ IgM, ↓ MBCs (CD20+ CD27+), ↓ NKCs, ↓ monocytes, lymphopenia, eosinophilia, ↓ CTLs, ↓ effector-memory CD8+ T cells, ↓ Treg, ↑ DN *αβ* T cells	[42]

*Note:* Recurrent infections, especially recurrent respiratory tract infections, were similar clinical features across various genetic categories and are not listed in the table. Treg, regulatory T (CD4 + CD25 + FoxP3+); Tfh, follicular helper T (CD4 + CD45RO + CXCR5+); TCM, central memory T cells.

Abbreviations: APDS, activated phosphoinositide 3-kinase delta syndrome; CMV, cytomegalovirus; CNS, central nervous system; DD, developmental delay; GHD, growth hormone deficiency; GN, glomerulonephritis; LAD, lymphadenopathy; MTCs, memory T cells; PSC, primary sclerosing cholangitis; RBI, recurrent bacterial infections; RTEs, recent thymic emigrants; UTIs, urinary tract infections.

**Table 3:** Monogenetic defects of nuclear molecules in CVID.

**Genetic defect**	**Disease**	**Mutation type**	**Inheritance pattern**	**Disease onset**	**Clinical manifestations**	**Immunological features**	**Ref**
NFKB1	NFKB1 deficiency	LOF	AD	Childhood to adulthood	AIE, ITP, AIT, AIN, AIHA, ILD, alopecia, PG, NRH hepatosplenomegaly, lung fibrosis, non-Hodgkin's lymphoma, thyroiditis autoimmune cytopenia	↓ or nl IgG, nl or ↓ IgA, nl or ↓ IgM, ↓ MBCs	[43]
NFKB2	NFKB2 deficiency	LOF	AD	Neonatal-onset to childhood	ITP, alopecia, endocrinopathies, inflammation of CNS	↓ IgG, ↓ IgM, ↓ IgA, ↓ post-vaccination antibody levels, ↓ B cells, ↓ switched MBCs, absent plasma cells, ↑ CD4/CD8 ratio, ↓ NKCs or NK toxicity	[44–46]
PTEN	PTEN deficiency	LOF	AD	Neonatal-onset	AI, developmental delay, LPD	nl IgG, ↓ IgA, ↓ IgM, ↓ MBCs, ↓ switched MBCs (CD27 + IgM–IgD–)	[47]
CTNNBL1	CTNNBL1 deficiency	NA	AR	NA	Autoimmune cytopenia, hyperplastic germinal centers	↓ IgG, ↓ IgM, ↓ IgA, ↓ MBCs, defective Ig CSR and SHM, immune-mediated thrombocytopenia	[48]
TRNT1	TRNT1 deficiency	LOF	AR	Neonatal-onset	Syndromic disorder with sideroblastic anemia and primary B-cell immunodeficiency, periodic fevers, and developmental delay (SIFT)	B-cell deficiency and hypogammaglobulinemia	[49, 50]
IRF2BP2	IRF2BP2 deficiency	GOF	AD	Childhood	AI, T1DM	↓ IgG, ↓ IgA, ↓ IgM, ↓ post-vaccination antibody levels, nl total B cells, ↓ MBCs, nl total/Th/CTLs T cells, ↓ or nl NKCs	[51]
SEC61A1	SEC61A1 deficiency	DN	AD	Neonatal-onset	GIs, sinusitis, pneumonia	↓ IgG, ↓ IgM, ↓ IgA, and ↓ post-vaccination antibody levels, nl B and T-cell subpopulations	[52]
ATP6AP1	ATP6AP1 deficiency	ND	XL	NA	RBI, low copper, hepatomegaly, cognitive impairment	Hypogammaglobulinemia, ↑ or nl NBCs, ↓ switched MBCs, ↓ post-vaccination antibody levels, nl or ↑ mature B, ↓ switched MBCs, ↓ central and effector CD8+ cells	[53]
IKZF1	IKAROS deficiency	LOF	AD	Childhood to adulthood	Skin infections, pneumonia, GIs, ALL, aphthous ulcers, AI	↓ IgG, ↓ or nl IgM and IgA, ↓ post-vaccination antibody levels, nl or ↓ total B cells, nl or ↓ MBCs, ↓ or nl or ↑ NKCs, nl or ↑ total T cells, ↓ or nl or ↑ Th cells, nl or ↑ CTLs	[54]

*Note:* Recurrent infections, especially recurrent respiratory tract infections, were similar clinical features across various genetic categories and are not listed in the table.

Abbreviations: ALL, acute lymphoblastic leukemia; ARF, acute respiratory failure; CSR, class-switch recombination; DN, dominant-negative; LGL, large granular lymphocytic; MS, multiple sclerosis; NBCs, naive B cells; NRH, nodular regenerative hyperplasia; NTCs, naive T cells; PG, pyoderma gangrenosum; SHM, somatic hypermutation; T1DM, Type 1 diabetes mellitus.

## Data Availability

The authors have nothing to report.
